# Use of transient elastography for hepatic steatosis and fibrosis evaluation in patients with subclinical hypothyroidism

**DOI:** 10.20945/2359-4292-2023-0477

**Published:** 2024-08-09

**Authors:** Milena Tauil Auad Noronha Santos, Cristiane Alves Villela-Nogueira, Nathalie Carvalho Leite, Patrícia de Fátima dos Santos Teixeira, Marcus Vinicius Leitão de Souza

**Affiliations:** 1 Universidade Federal do Rio de Janeiro Hospital Universitário Clementino Fraga Filho Divisão de Endocrinologia Rio de Janeiro RJ Brasil Divisão de Endocrinologia, Hospital Universitário Clementino Fraga Filho, Universidade Federal do Rio de Janeiro, Rio de Janeiro, RJ, Brasil; 2 Universidade Federal do Rio de Janeiro Hospital Universitário Clementino Fraga Filho Divisão de Hepatologia Rio de Janeiro RJ Brasil Divisão de Hepatologia, Hospital Universitário Clementino Fraga Filho, Universidade Federal do Rio de Janeiro, Rio de Janeiro, RJ, Brasil

**Keywords:** Transient elastography, subclinical hypothyroidism, nonalcoholic fatty liver disease, fatty liver, metabolic diseases

## Abstract

**Objective:**

To evaluate the association between subclinical hypothyroidism and hepatic steatosis and fibrosis using the noninvasive diagnostic methods transient hepatic elastography (TE) and controlled attenuation parameter (CAP) in patients with subclinical hypothyroidism.

**Subjects and methods:**

This was a cross-sectional study including women with confirmed spontaneous subclinical hypothyroidism and an age- and body mass index (BMI)-matched control group without thyroid disease or circulating antithyroperoxidase (anti-TPO) antibodies. Exclusion criteria were age > 65 years, thyroid-stimulating hormone (TSH) > 10.0 mIUI/L, BMI ≥ 35 kg/m^2^, diabetes, or other chronic liver diseases. Liver stiffness was classified according to TE values (in kPa) and ranged from absence of fibrosis (F0) to advanced fibrosis (F3). Hepatic steatosis was classified according to CAP values (in dB/m) and ranged from low-grade (S1) to advanced (S3) steatosis.

**Results:**

Of 68 women enrolled, 27 were included in the subclinical hypothyroidism group and 41 in the control group. Advanced steatosis (S3) was more frequent in the subclinical hypothyroidism group (25.9% *versus* 7.3%, respectively, p = 0.034). Circulating anti-TPO was an independent factor associated with advanced steatosis (odds ratio 9.5, 95% confidence interval 1.3–68.3). In multiple linear regression analysis, TE values (which evaluated fibrosis) correlated negatively with free thyroxine levels.

**Conclusion:**

The results of this study strengthen the hypothesis that hepatic steatosis is associated with autoimmune (positive anti-TPO) subclinical hypothyroidism, independently from BMI. However, subclinical hypothyroidism alone does not appear to be associated with a significantly increased risk of hepatic fibrosis.

## INTRODUCTION

Metabolic dysfunction-associated steatotic liver disease (MASLD) is the latest term for describing steatotic liver disease associated with metabolic syndrome and is currently the most prevalent chronic liver condition (1). It encompasses a spectrum that ranges from simple steatosis to nonalcoholic steatohepatitis with fibrosis. Aside from concerns regarding its progression to cirrhosis and hepatocellular carcinoma, MASLD is also associated with an increased cardiovascular risk (2-4), which can overlap with the risk factors commonly associated with metabolic syndrome.

Subclinical hypothyroidism affects nearly 10% of all adults. Its treatment is primarily recommended for asymptomatic patients with TSH levels > 10 mIU/L, primarily due to evidence of an association between subclinical hypothyroidism and cardiovascular diseases, particularly stroke and coronary heart disease (5). Indeed, meta-analyses of multiple clinical trials have established a link between subclinical hypothyroidism and the aforementioned cardiovascular diseases. However, there are conflicting data regarding treatment benefits, with a greater amount of data supporting treatment for patients younger than 65 years (6). Abnormal lipid levels combined with endothelial dysfunction may be the main factors contributing to the observed elevated cardiovascular risk in subclinical hypothyroidism, but this association may be influenced by mechanisms other than the established risk factors (7).

While MASLD is generally regarded as the hepatic manifestation of metabolic syndrome, not all patients achieve MASLD remission, even after intense lifestyle modifications to improve metabolic syndrome – particularly those with advanced disease (8,9). This underscores the importance of gaining a deeper understanding of the MASLD pathophysiology.

Thyroid dysfunction may be an additional factor contributing to MASLD emergence. Indeed, the association of MASLD with overt hypothyroidism is well-documented and occurs independently from the individual's body mass index (BMI) (10,11). While thyroid hormone plays a crucial role in regulating metabolic rate, it is unclear whether treating elevated serum TSH levels with levothyroxine supplementation yields any benefit in terms of MASLD (12). Until definitive answers become available, identifying whether subclinical hypothyroidism has any correlation with MASLD could provide valuable information for clinical practice, as the primary motivation for treating this condition is often related to improving cardiovascular risk.

Transient hepatic elastography (TE) is a point-of-care, noninvasive method for diagnosing liver fibrosis (13). The use of TE combined with a controlled attenuation parameter (CAP) software – a tool for estimating liver steatosis – has been shown to be an accessible and valuable noninvasive method for identifying patients at a higher risk of chronic liver disease (14-16).

Based on these considerations, the aim of this study was to evaluate the usefulness of TE and CAP in evaluating hepatic steatosis and fibrosis in patients with subclinical hypothyroidism.

## SUBJECTS AND METHODS

### Study population

This was a cross-sectional study of a convenience sample of nonpregnant female patients aged 30-65 years recruited from an outpatient clinic. The inclusion criterion was a diagnosis of spontaneous subclinical hypothyroidism, determined by two serum TSH measurements between 4.0 mIU/L and 10 mIU/L, sampled 2 months apart, along with serum free thyroxine (fT4) levels within the normal range. For comparative analyses, the study also included a control group without thyroid disease, confirmed by normal thyroid function tests and negative antithyroid peroxidase (anti-TPO) antibodies, and with demographic characteristics similar to those in the subclinical hypothyroidism group.

The exclusion criteria were a BMI ≥ 35 kg/m^2^, diabetes, other chronic liver diseases (excluded by a negative test for hepatitis virus and also a negative HIV test), alcohol consumption > 20 g in the previous 5 years, and use of any medication with potential interference with thyroid function or associated with hepatic steatosis.

The participants underwent a complete physical examination and blood sample collection for biochemical analysis and thyroid function assessment. They were subsequently evaluated with TE and CAP (FibroScan 502 Touch; Echosens, Paris, France), carried out by a trained specialist. The assessor was blinded to the participants’ thyroid status. Reliability criteria for the exam were applied as recommended by the manufacturer.

The study protocol was approved by the Ethics Committee of the *Hospital Universitário Clementino Fraga Filho* (HUCFF/UFRJ, Rio de Janeiro, Brazil) with a Certificate of Presentation for Ethical Appreciation (CAAE) number 13748014.0.0000.5257 (number 009744/2014). The study was conducted in accordance with the Declaration of Helsinki on human research studies. Informed consent was obtained from all participants.

### Thyroid function assessment

Thyroid function assessments included measurement of TSH, fT4, and anti-TPO antibody levels in two serum samples. The measurements were obtained using an electrochemiluminescence immunoassay conducted on Immulite 2000 equipment (Diagnostic Products Corporation, Los Angeles, CA, USA). The reference ranges were 0.4-4.0 mIU/L for TSH, 0.8-1.9 ng/dL for fT4, and < 35 IU/mL for anti-TPO antibodies. The diagnosis of subclinical hypothyroidism followed the criteria recommended in the Brazilian consensus (17).

### Assessment of hepatic steatosis

Hepatic stiffness and steatosis were assessed using TE and CAP, respectively, performed on the FibroScan 502 Touch equipment, as mentioned. A single operator performed the TE on the right hepatic lobe, between the sixth and seventh intercostal spaces, while the participants lay flat on their backs with their right arms in maximum abduction. At least 10 reliable measures were obtained with a maximum interquartile range of 30%. All patients fasted for 3 hours before the examination. Either the M or XL probe was used, as per the manufacturer's instructions.

The reliability of liver stiffness measurements was defined according to the criteria by de Lédinghen and cols. (15). The values were expressed in kilopascal (kPa) units, and could vary between 1.5 and 75 kPa. The CAP values were expressed as decibels per meter (dB/m) and could vary between 100 and 400 dB/m.

Liver steatosis was defined according to de Lédinghen and cols. (15), using the following CAP values: 215-251 dBm/min, steatosis (S1); 252-295, moderate steatosis (S2); and ≥ 296 dBm/min, severe steatosis (S3). These values correspond to the percentages of hepatocytes with fat, where S0 is < 11%, S1 is 11%-33%, S2 is 34%-66%, and S3 is > 66%.

To quantify liver fibrosis, TE assesses the stiffness of the liver parenchyma. According to Wong and cols. (16), a TE < 7.9 kPa indicates a high negative predictive value for advanced fibrosis, while a value > 9.6 kPa suggests a high positive predictive value for advanced fibrosis (F ≥ F3).

### Statistical analysis

The software SPSS for Windows, Version 21.0 (SPSS Inc., Chicago, IL, USA) was used for the statistical analysis. The Mann-Whitney test was used to compare continuous variables between the two groups, which were described as mean ± standard deviation (median) values. The chi-square or Fisher's exact test was used to compare categorical variables, which were described as frequencies. Correlations between continuous variables were analyzed using Spearman's correlation coefficient in bivariate analysis.

Binary logistic regression analysis was performed in different models considering advanced steatosis as the dependent variable for the assessment of independent variables related to it. The variables included in Step 1 of Model 1 were BMI, age, and subclinical hypothyroidism. Step 2 of Model 1 also included the presence of circulating anti-TPO antibodies. A second model (Model 2) was fit retaining all variables from Model 1 but changing subclinical hypothyroidism to serum TSH as a continuous variable. Finally, we tested a model including the following variables potentially related to advanced steatosis: subclinical hypothyroidism, presence of anti-TPO antibodies, obesity, metabolic syndrome, and age > 50 years.

Multiple linear regression was performed to test whether the correlations between serum TSH, fT4, and TE remained after adjusting for age, BMI, and anti-TPO antibodies as confounding variables. Step 1 evaluated only TSH, and Step 2 also included fT4 in the model.

The statistical significance was set at p < 0.05. However, p values between 0.05 and 0.10 were considered indicative of borderline associations and were included in the discussion of the results.

## RESULTS

The study included 68 eligible participants with complete thyroid function evaluation, who underwent TE assessment. In all, 27 patients were included in the subclinical hypothyroidism group and 41 were included in the control group. Both groups were matched for age and BMI. Table 1 summarizes the baseline characteristics of the participants.

**Table 1:** Baseline characteristics of the study participants

Characteristics	Subclinical hypothyroidism group (n = 27)	Control group (n = 41)	P values
Age (years)	50.6 ± 9.4 (53)	47.6 ± 8.6 (46.0)	0.118
TSH level (mIU/L)	6.2 ± 1.5 (6.0)	1.6 ± 0.6 (1.6)	<0.001
fT4 level (ng/dL)	0.98 ± 0.2 (0.9)	1.06 ± 0.1 (1.0)	0.01
Body mass index (kg/m^2^)	28.9 ± 4.4 (30.0)	28.0 ± 5.0 (28.1)	0.257
Abdominal circumference (cm)	96.6 ± 11.4 (99.0)	92.4 ± 10.8 (92.0)	0.223
High blood pressure (% of patients)	41	25	0.15
Tobacco use (% of patients)	11.1	14.3	0.88
Anti-TPO + (% of patients)	41.0	0.0	<0.01

Abbreviations: Anti-TPO +, positive antithyroperoxidase antibodies; fT4, free thyroxine; TSH, thyroid-stimulating hormone.

The values of TE and CAP did not differ between groups, nor did the frequency of steatosis detected by CAP. However, advanced stages of liver steatosis were more frequent in the subclinical hypothyroidism group and were associated with thyroid status (odds ratio [OR] 3.5, 95% confidence interval [CI] 1.003-12.52, p = 0.034) (Table 2).

**Table 2:** Transient hepatic elastography and controlled attenuation parameter values in the subclinical hypothyroidism and control groups

	Subclinical hypothyroidism group (n = 27)	Control group (n = 48)	P values – OR (95% CI)
CAP (dB/m)	256.4 ± 47.3 (259.0)	242.9 ± 44.9 (238.0)	0.18
Frequency of detectable steatosis (%)	55.6	43.9	0.34-1.2 (0.78-2.05)
Frequency of advanced steatosis (S3) (%)	**25.9**	**7.3**	**0.034-3.5 (1.01-12.5)**
TE (kPa)	5.2 ± 1.7 (5.3)	4.6 ± 1.3 (4.3)	0.156
TE > 7.9 kPa (%)	3.7	2.4	0.762-0.7 (0.04-10.8)
TE > 9.6 kPa (%)	3.7	0	0.214-0.96 (0.9-1.1)

Abbreviations: CAP, controlled attenuation parameter; CI, confidence interval; OR, odds ratio; TE, transient hepatic elastography.

Bivariate analysis showed an association between severe steatosis and subclinical hypothyroidism, which was lost in the binary logistic regression analysis considering age and BMI as confounding variables (Table 3). Only BMI was independently associated with severe steatosis (OR 1.2, 95% CI 1.01-1.38) in this model. When anti-TPO positivity was added to the model (Step 2), a trend toward an association with severe steatosis was observed.

**Table 3:** Results of binary logistic regression analysis identifying independent variables associated with advanced steatosis

	Model 1				Model 2	
	Step 1 OR (95% CI)	P values	Step 2 OR (95% CI)	P values	OR (95% CI)	P values
BMI	1.2 (1.01-1.4)	0.043	1.2 (1.03-1.4)	0.046	1.2 (1.01-1.4)	0.046
Age	1.1 (0.97-1.2)	0.167	1.1 (1.0-1.2)	0.126	1.1 (1.0-1.2)	0.111
SCH or TSH	3.6 (0.7-17.6)	0.106	1.3 (0.1-9.8)	0.786	0.9 (0.7-1.4)	0.992
Anti-TPO +	N/A	N/A	7.8 (0.9-64.5)	0.057	9.5 (1.3-68.3)	0.024

Model 1 included the variables BMI, age, and SCH in Step 1 and circulating anti-TPO antibodies in Step 2. Model 2 retained all variables from Model 1 Step 2, replacing SCH with serum TSH level as a continuous variable. Abbreviations: anti-TPO +, presence of antithyroperoxidase antibodies; BMI, body mass index; CI, confidence interval; N/A, not applicable; SCH, subclinical hypothyroidism; TSH, thyroid-stimulating hormone.

Table 4 shows the results of another binary logistic regression model, confirming an independent association between circulating anti-TPO antibodies and severe steatosis.

**Table 4:** Results of bivariate linear regression analyzing independent variables associated with advanced hepatic steatosis, considering the presence of obesity and metabolic syndrome in the model

Variables	OR	95% CI	P values
Subclinical hypothyroidism	0.985	0.11-8.12	0.98
**Anti-TPO +**	**10.3**	**1.20-89.9**	**0.03**
Age > 50 years	1.96	0.38-10.1	0.42
Metabolic syndrome	1.69	0.28-10.3	0.57
Obesity	3.52	0.44-28.1	0.23

Metabolic syndrome was defined as the presence of at least three criteria defined by the National Cholesterol Education Program (NCEP) Adult Treatment Panel III (ATP III). Obesity was defined as a body mass index ≥ 30 kg/m^2^. Abbreviations: anti-TPO +, positive antithyroperoxidase antibodies; CI, confidence interval; OR, odds ratio.

Although the frequency of detectable or advanced fibrosis in the subclinical hypothyroidism group was not significantly different from that in the control group (Table 2), liver stiffness correlated positively with serum TSH level and negatively with serum fT4 level in the bivariate analysis (Table 5). Table 5 also shows the results of the bivariate (Spearman's) correlation analysis of TSH and fT4 levels with CAP and TE values, steatosis grade, and fibrosis stage.

**Table 5:** Results of bivariate analysis assessing the correlation of TSH and free T4 levels with liver steatosis (evaluated by controlled attenuation parameter) and liver stiffness (evaluated by transient hepatic elastography)

	TSH		Free T4	
	r_s_	P value	r_s_	P value
**CAP (mean)**	0.285	0.009	-0.038	0.378
**Steatosis grade**	0.285	0.009	-0.107	0.190
**TE (kPa)**	0.278	0.011	-0.251	0.02
**Fibrosis stage**	0.171	0.08	-0.06	0.30

Abbreviations: CAP, controlled attenuation parameter; TE, transient hepatic elastography; rs, Spearman's correlation coefficient.

On multiple linear regression evaluating whether the correlation between higher TSH and fT4 levels with liver stiffness was maintained in multivariate analysis (Table 6), only fT4 correlated negatively with liver stiffness in Model 2.

**Table 6:** Multiple linear regression evaluating possible variables influencing liver stiffness assessed by transient hepatic elastography*

	Model 1			Model 2		
	Beta (95% CI)	t	P value	Beta (95% CI)	t	P value
BMI	0.014 (-0.0-0.03)	2.07	0.042	0.012 (-0.0-0.1)	1.73	0.088
Age	0.005 (-0.003-0.01)	1.28	0.204	0.005 (-0.002-0.01)	1.54	0.128
TSH	0.024 (-0.01-0.05)	1.65	0.103	0.022 (-0.4-0.3)	1.53	0.131
Anti-TPO +	-0.068 (-0.3-0.12)	-0.71	0.478	-0.129 (-0.3-0.1)	-1.32	0.191
Free T4	N/A	N/A	N/A	-0.473 (-0.9-0.0)	-2.04	0.045

* Log transformed. Abbreviations: anti-TPO +, positive antithyroperoxidase antibodies; BMI, body mass index; N/A, not applicable; TE, transient hepatic elastography.

## DISCUSSION

A relationship between thyroid function and liver steatosis was initially established in animal studies. These studies demonstrated a reduction in liver steatosis with the use of agonist agents targeting hepatic thyroid receptors (18,19). In humans, a randomized clinical trial has shown the benefits of levothyroxine supplementation in patients with subclinical hypothyroid and dyslipidemia (20).

The present study confirms the findings from earlier studies identifying that being overweight is an independent risk factor for advanced steatosis (21). It also revealed that subclinical hypothyroidism is associated with liver steatosis, albeit with a weaker association when metabolic factors were included in the regression model. Importantly, the present study is the first to have shown an association between liver steatosis and autoimmune subclinical hypothyroidism, identified by the presence of anti-TPO antibodies.

Notably, TSH itself can directly impact hepatocyte function through the TSH receptor signaling pathway (22-24). The results of the bivariate analysis in the present study indicated a positive correlation between liver stiffness (identified by TE) and fibrosis stage with serum TSH level and a negative and independent correlation of fT4 level with liver stiffness. These findings contrast with those of a previous study, which identified an association between liver fibrosis and subclinical hypothyroidism (25). The discrepancy between both studies may be attributed to the characteristics of our study group, which was primarily composed of young overweight women, in whom subclinical hypothyroidism may represent a subtle additional risk factor for steatosis. In the final model of the multiple regression analysis, obesity emerged as an independent factor associated with steatosis, as already established in previous studies.

Data suggesting an association between subclinical hypothyroidism and liver steatosis may be confounded (11,26-31) when compared with data that also include overt hypothyroidism (32-34). The findings of the present study are aligned with those from previous results, including trials and meta-analyses, investigating the association between subclinical hypothyroidism and hepatic steatosis (25,35). The mechanisms underlying this association likely involve lipophagy mediated by thyroid hormone receptors, which affects fatty acid oxidation and the removal of liver lipids. The process, thus, leads to lipid accumulation in the liver and the subsequent development of steatosis (36,37).

In the present study, liver stiffness assessed by TE (reflecting fibrosis stage) showed a positive correlation with serum TSH level in the bivariate analysis. However, serum fT4 level exhibited a negative and independent association with liver stiffness and fibrosis. In contrast with this finding, a large cohort study has identified an association between liver fibrosis and subclinical hypothyroidism, as mentioned earlier (25). It is important to note that our patient population consisted of young individuals, and it is well-established that fibrosis is more prevalent among individuals in the fifth and sixth decades of life. The cross-sectional design of the present study limits our ability to understand the long-term development of fibrosis in our patients. It is possible that the lower fT4 levels may have resulted from hepatic fibrosis, but the study's cross-sectional design is limited in addressing this issue. Obesity and metabolic syndrome are potentially significant confounding factors in studies that have concluded that lower T4 and higher TSH levels (even when within the normal range) may be associated with MAFLD and liver fibrosis (38-40). In our study cohort, both BMI and metabolic syndrome were identified as independent factors associated with steatosis. Surprisingly, the subclinical hypothyroidism group exhibited the same association when circulating anti-TPO antibodies were present, despite the small number of patients with these antibodies (41% of all patients with subclinical hypothyroidism). While previous hypotheses have primarily centered around the possibility of direct autoimmune liver injury as the mechanism through which subclinical hypothyroidism may influence liver steatosis (41), it is worth considering that anti-TPO antibodies may serve as a marker for genuine thyroid dysfunction. Many cases of subclinical hypothyroidism may represent an adaptive adjustment of the thyroid axis in response to nonthyroidal illness. Therefore, the association between subclinical hypothyroidism and anti-TPO antibodies may reflect a "true" subclinical dysfunction that could influence liver steatosis, or, as previously suggested, these antibodies may impact liver fibrosis by indicating immune aggression.

The primary challenge in assessing liver steatosis and fibrosis is the disparity between serum biochemical tests and noninvasive diagnostic tools with the highly invasive procedure of liver biopsy. Many studies draw conclusions based on regular ultrasound images and indirect biochemical parameters to assess liver steatosis, which often do not align with findings from liver biopsy studies (42). However, studies show a stronger correlation between liver biopsy results with TE compared with other noninvasive methods (43). The only available study specifically analyzing patients with hypothyroidism by using TE has yielded results similar to ours, with the main distinction being the association of fibrosis with subclinical hypothyroidism, as previously discussed (25). In light of these ongoing controversies and the cost-effectiveness of TE as a noninvasive diagnostic tool, TE could provide valuable clinical insights, particularly in young and nonobese patients with subclinical hypothyroidism.

The limitations of the present study must be recognized. The limited sample size, the cross-sectional design, and the absence of a drug intervention represent inherent constraints that warrant caution when definitive conclusions are drawn from the study. Although well-validated in MASLD, TE is not the gold-standard method for identifying steatosis or liver fibrosis in MASLD. However, it would not be feasible to perform liver biopsy in a healthy population with subclinical hypothyroidism, since this is an invasive method and is also prone to limitations. Hence, TE, being a noninvasive tool, is valuable for the risk stratification of liver diseases, with good accuracy. Future research, particularly with longitudinal approaches focusing on individuals with positive anti-TPO antibodies, may offer a more comprehensive understanding of the observed correlations. Given the ongoing debate surrounding the present topic, the discrepancies underscore the complexity of this subject and highlight the need for further studies to clarify the underlying mechanisms and factors contributing to the conflicting outcomes. Despite these limitations, the present study provides valuable insights into the existing body of knowledge on the present topic. The study findings reinforce the hypothesis of a link between advanced liver steatosis and subclinical hypothyroidism, potentially mediated by autoimmunity – particularly subclinical hypothyroidism with positive anti-TPO antibodies – and independent from BMI. Additionally, parameters associated with fibrosis were observed to correlate with lower fT4 levels. However, it is important to note that the absence of an association between subclinical hypothyroidism and liver fibrosis may have been influenced by the small number of patients with subclinical hypothyroidism in the present study.
